# The Architecture of Early Childhood Sleep Over the First Two Years

**DOI:** 10.1007/s10995-022-03545-9

**Published:** 2022-12-31

**Authors:** Sonia Marie Lenehan, Leanna Fogarty, Cathal O’Connor, Sean Mathieson, Geraldine B. Boylan

**Affiliations:** INFANT Research Centre, University College Cork, Cork, Ireland

**Keywords:** Infant sleep, NREM, REM, Term infants, Sleep deprivation, Sleep–wake cycle, Neurodevelopment, Early childhood

## Abstract

**Introduction:**

The architecture and function of sleep during infancy and early childhood has not been fully described in the scientific literature. The impact of early sleep disruption on cognitive and physical development is also under-studied. The aim of this review was to investigate early childhood sleep development over the first two years and its association with neurodevelopment.

**Methods:**

This review was conducted according to the 2009 PRISMA guidelines. Four databases (OVID Medline, Pubmed, CINAHL, and Web of Science) were searched according to predefined search terms.

**Results:**

Ninety-three studies with approximately 90,000 subjects from demographically diverse backgrounds were included in this review. Sleep is the predominant state at birth. There is an increase in NREM and a decrease in REM sleep during the first two years. Changes in sleep architecture occur in tandem with development. There are more studies exploring sleep and early infancy compared to mid and late infancy and early childhood.

**Discussion:**

Sleep is critical for memory, learning, and socio-emotional development. Future longitudinal studies in infants and young children should focus on sleep architecture at each month of life to establish the emergence of key characteristics, especially from 7–24 months of age, during periods of rapid neurodevelopmental progress.

## Significance Statement

Human neonates and infants spend most of the time asleep. Despite this, there is a lack of knowledge surrounding sleep, its function, and its relationship with neurodevelopment. Ninety-three studies were included in this review. Together they highlight the role sleep plays in social and emotional development as well as learning and memory. Most information on early childhood sleep relates to sleep in the first six months of life and future research should focus on the role that sleep plays in neurodevelopment between 7 and 24 months of age.

## Introduction

The architecture of sleep changes markedly over the first two years of age. Despite increasing research interest, gaps remain in our knowledge of infantile sleep, its precise function, and the impact of sleep disruption in infancy and early childhood on cognitive and physical development. Few recommendations exist to educate healthcare providers and parents of young children about the importance of sleep and its impact on overall health (Mukherjee et al., 2015). The majority of research on neonatal sleep generally includes older children, adults, and animal models. Sleep studies focusing on normal sleep in early childhood are necessary to ensure greater understanding of the role sleep plays in cognitive development (Jiang, 2019; Tham et al., 2017).

Neonatal sleep is characterized by Active Sleep (AS), Quiet Sleep (QS), and Indeterminate Sleep (IS). Active Sleep is also called Paradoxical Sleep (Samson-Dollfus et al., 1983); it is characterised by rapid eye movements, irregular breathing, body and limb movements, low voltage electroencephalography (EEG), and high variability in heart rate (Barbeau & Weiss, 2017; Mirmiran et al., 1993). AS is believed to play a role in the maturation of the central nervous system (CNS) and facilitate growth and development (Denenberg & Thoman, 1981; Mirmiran et al., 1993). Evidence of the role of AS in brain development comes from research using animal models (Bertelle et al., 2005; Mirmiran, 1995; Mirmiran et al., 1983, 1988, 1993). QS is characterised by reduced eye movements, regular breathing, decreased body movements, slow wave activity on the EEG, and low variability in the heart rate (Barbeau & Weiss, 2017; Mirmiran et al., 1993). It is presumed that QS plays a role in energy maintenance, the release of growth hormones (Bertelle et al., 2005) and has a restorative function (Mirmiran, 1995). After two months of age AS becomes Rapid Eye Movement (REM) Sleep, and QS becomes non-Rapid Eye Movement (NREM) Sleep. When elements of both AS and QS are present, it is described as indeterminate sleep (Bertelle et al., 2005; Korotchikova et al., 2009). This stage is considered a transitional stage or measure of immature sleep (Louis et al., 1997).

Sleep structure in early childhood is very different to sleep in adulthood. At birth, an ultradian rhythm dominates, and infants and young children spend a greater period of time asleep in a 24 h period and have different EEG patterns to older children and adults during sleep (Barry, 2020). Rapid changes occur in sleep structure within the first 12 weeks and continue throughout childhood (Dittrichova, 1966). Throughout this period, sleep is restructuring and reorganising in parallel with rapid brain growth and neuroplasticity (Bes et al., 1988). This is best seen on EEG, where patterns of sleep undergo swift changes especially during the first few months, e.g. the disappearance of *tracé alternant*, the appearance of sleep spindles (Jenni et al., 2004), and the disappearance of focal sharp waves (Ellingson & Peters, 1980).

Sleep has been associated with learning and memory and emotional regulation. However the role of sleep during brain development in early childhood is not well known. (Jiang, 2019). Understanding sleep and its role in development is necessary to;

1. Ensure caregivers have the necessary information to support normative development of infant and young children’s sleep (Paavonen et al., 2020)
2. Understand the development of normative sleep and the range of normative sleep variables (Jiang, 2019; Paavonen et al., 2020), and
3. Investigate the potential of sleep as a target for early intervention to optimize development (Tham et al., 2017).

The aim of this narrative review is to discuss available literature relating to sleep development and the impact of sleep on neurodevelopment in the first two years and to identify gaps in the scientific literature at this critical stage of neurodevelopment.

## Methods

### Search Strategy

The search strategy for this narrative review was conducted according to the 2009 Preferred Reporting Items for Systematic Reviews and Meta-analysis (PRISMA) guidelines (Liberati et al., 2009). Inclusion and exclusion criteria were established, databases were identified, search terms and data to be collected were agreed upon by authors SL and LF. In May 2021, SL searched four databases (OVID Medline, Pubmed, CIMAHL, and Web of Science). The full search strategy is shown in Table 1. No limitations were applied to the publication years. The papers were extracted using EndNote™ Version X9. Duplicates were removed. Extracted papers were included or excluded based on title and abstract. Full articles were screened for inclusion based on the below criteria. The reference lists of all relevant articles were checked manually for papers potentially missed by the search.

**Table 1:**
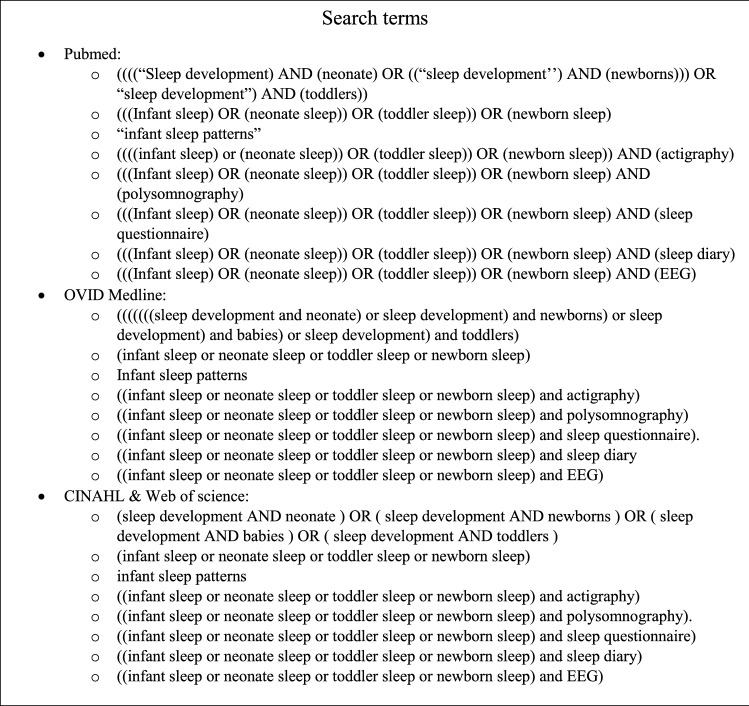
Search strategy for the four databases used in the review

### Inclusion Criteria

Studies were included if:

- Participants were healthy infants and young children up to the age of 2 years.
- More than five subjects were included.
- Objective (polysomnography, actigraphy) or subjective (self-reported) measures of sleep were recorded, such as sleep duration, night-waking, sleep latency, longest sleep period, daytime naps, sleep architecture, and sleep efficiency.
- Sleep variables and neurodevelopmental or cognitive-developmental outcomes were reported.

### Exclusion Criteria

Studies were excluded if:

- Participants were born preterm (< 37 weeks’ gestational age) or admitted to a Neonatal Intensive Care Unit (NICU).
- Cohort had a mean age > 24 months.
- Behavioural interventions were performed.
- Studies were validation of methodology or computational models, and did not contribute information on sleep.
- Cohorts were duplicated, unless assessed at different time points.
- Sleep data or development data were not reported in results.
- Type of publication was a review article, book chapter, dissertation, or conference abstract.
- No English translation was available.

### Data Collection

Author, year, sample size, assessment age, sleep variables reported in the studies, and developmental assessments were collected. Reported hours of sleep were collected from each article for 24 h and/or 12 h for; total sleep time (TST), AS, QS, IS, REM, and NREM. The weighted mean was calculated for each variable that had data available using the formula: weighted mean = sum of (number x weighting factor)/sum of all the weights.

## Results

**Fig. 1:**
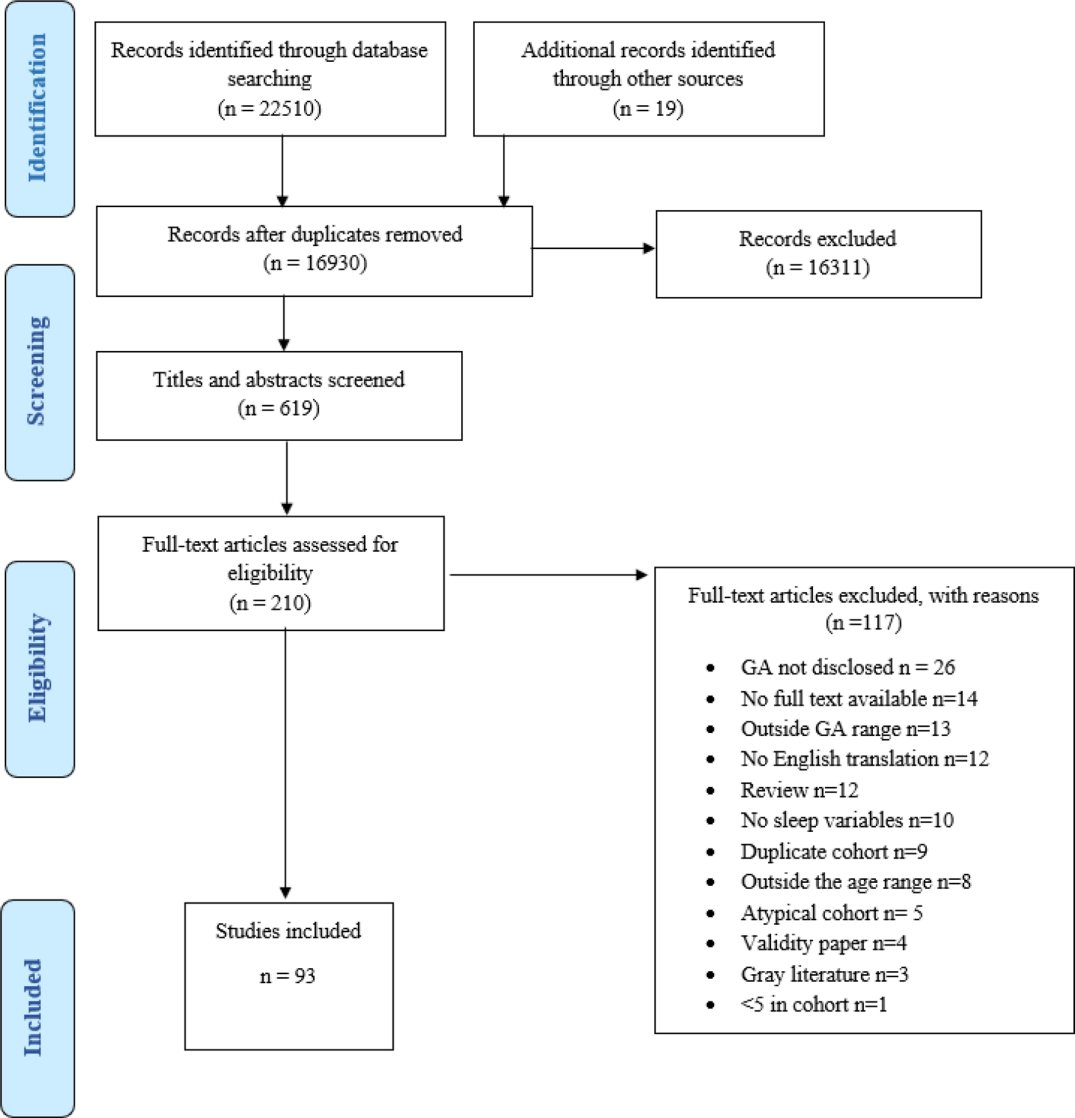
Flow chart indicating the results of the database search with inclusions and exclusions. Template of the flowchart was taken from Liberati et al. (2009) *GA* Gestational Age

**Fig. 2:**
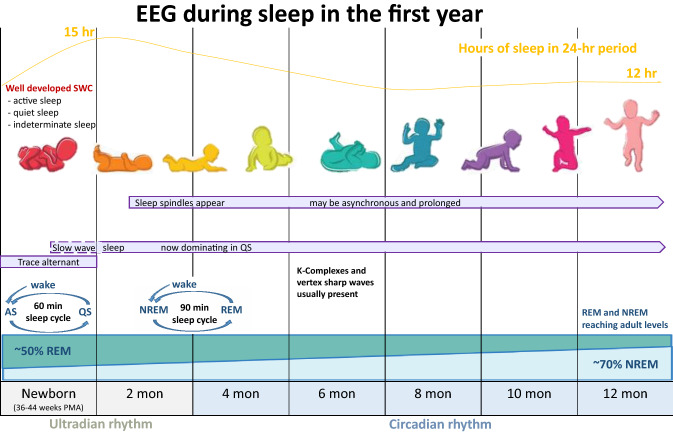
Visual summary of the development of sleep in the first year; showing the development of sleep milestones in the EEG

Results from the search strategy are shown in Fig. 1. Ninety-three studies assessed sleep in approximately 90,000 subjects. Table 2 lists the characteristics of the studies, including sample size (male:female), cohort age, sleep variables, sleep assessment tools, and development assessment. Tools included EEG, actigraphy, questionnaires, and sleep diaries. Most studies used one tool to assess sleep, eighteen used two, and four used three tools. Forty-one studies assessed sleep using one assessment in the first year, 31 studies used multiple assessments over the first year, 6 studies used one assessment in the second year, and two studies used multiple studies over the second year. Four studies used one assessment with the cohort ranging over the first two years. Table 3 shows the weighted means of daytime sleep, night-time sleep, and TST over 24 h. Figure 2 provides a visual summary of the development of sleep in the first year, showing the appearance and disappearance of *tracé alternant*, the appearance of slow wave sleep and spindles, and the number of hours of sleep in a 24 h period.

**Table 2:** Characteristics of included studies

Author	Category	Sample size (M:F)	Age at assessment	Sleep assessment	Sleep variable	Developmental assessments
(Acebo et al., 2005)	Sleep Patterns	169 (84:85)	12, 18, & 24, months	Sleep diaries, Actigraphy	Diary: Bedtime, Rise time, TIB, Reported Wake minutes, Nap duration, No. of Naps. Actigraphy: Sleep start time, Sleep end time, Sleep period, Wake mins, SE, Mins of longest continuous sleep, Mean activity	
(Adams et al., 2019)	Sleep development	24 (14:10)	6, 15, & 24 weeks	Actigraphy, BISQ	Actigraphy: Night-time sleep interval duration, Total daytime nap duration, Total 24 h sleep duration, Sleep maintenance efficiency, Avg. nap duration, No. naps per day,	
(Anders & Roffwarg, 1973)	Sleep deprivation	17 (6:11)	24–96-h-old	EEG	% REM sleep, NREM sleep and awake	
(Anders, 1978)	Sleep patterns	32 2 months (16:16), 9 months (19:17)	2 & 9 months	Time-lapse video	Total recording period, out of crib, Awake, QS, AS	
(Ashton, 1971)	Behavioural sleep cycles	22 (12:10)	Avg. age 72.8 h postpartum	Observation & polygraph	Crying, Activity, Eyes open, Mean duration of Alert state, AS & QS	
(Atun-Einy & Scher, 2016)	Sleep disruption	20 (14:6)	7 & 11.5–12 months	Actigraphy	SE & Night waking	
(Bamford et al., 1990)	Sleep patterns	174	6, 13, 26, & 52 weeks	Diary, Questionnaire, & Interviews	Mean number of episodes of sleep, TST	
(Becker & Thoman, 1981)	REM storms	Group 1: 15 (8:7) Group 2: 14 (6:8)	2, 3, 4, & 5 weeks. 3, 6 & 12 months	Sleep observation	AS, QS, S-W transition. Amount of time slept. Level of REM activity on a 4-point scale	Bayley Scales of Mental Development
(Bes et al., 1988)	Sleep development	48	1–54 weeks	EEG	Qualitative evaluation, Range of the EEG parameter, Range differences within the night, Recurrence time of EEG synchronisation and desynchronisation	
(Bernier et al., 2010)	Sleep development	60 (36:24)	12–13, 18, & 26 months	Sleep diary	Total sleep duration, % of total sleep occurring at night (7am—7 pm), and sleep fragmentation (no. of night-awakenings)	EF assessment, Mental Development Index of the Bayley Scales of Infant Development, MacArthur Communicative Developmental Inventory
(Blair et al., 2012)	Sleep development	11,478 (5922:5556)	6, 18 months	Questionnaires	Night-time sleep duration, Daytime sleep, Total daily sleep, No. of night-awakenings	
(Carroll et al., 1999)	Sleep development	47	2 days old	Motility Monitoring System	AS, QS, A-Q transitional sleep, S-W transition, Wake, Periods out-of-the-crib	
(Cheour et al., 2002)	Learning and Memory	45	2–7 days old	Mismatch Negativity (MMN)	MMN recordings to analyse the responses to vowel sounds	
(Coons & Guilleminault, 1984)	Sleep development	30	3 & 6 weeks, 3 & 6 months	EEG, EOG, EMG, ECG	Longest sleep period, longest wake period, Spindles, Delta waves, REM, NREM, TST of NREM sleep spindles, Efficiency of a sleep stage, longest period of uninterrupted sleep analysed for duration, Type of onset, Position in 24 h, Stage sequence and efficiency	
(Corsi-Cabrera et al., 2020)	Sleep development	60	41–45 weeks post-conception	Polysomnography	Wakefulness, AS, QS, Transitional sleep, Quantitative EEG analysis	
(D'Atri et al., 2018)	Sleep development	39 (25:14)	0–48 months 0–3 n = 14, 4–12 n = 7, 13–24 n = 11,	Polysomnography	Total sleep period, TST, QS/NREM, AS/REM, % WASO, Sleep spindle detection	
(Denenberg & Thoman, 1981)	Sleep development	21	2–5 weeks	Sleep observed by researchers	Waking Active, Quiet Alert, Fuss or Cry, Drowse or Transition, AS, QS	
(Dittrichová et al., 1972)	Sleep development	19	2, 4, 6, 9, 12, 16, 20, & 24 weeks of age	EEG, EMG, EOG	Duration of the REM epoch, Frequency of REMs, Intervals between single REMs	
(Eaton-Evans & Dugdale, 1988)	Sleep–wake patterns	132	Monthly from 1–12 months	Interviews	Night waking, Factors associated with night waking	
(Eiselt et al., 2001)	Sleep development	6	3–8 days old	EEG	Sleep states	
(Ellingson & Peters, 1980)	Sleep development	17 (5:12)	8–9 months	EEG	Wakefulness, drowsiness (D), QS/SWS, AS/REMS, or IS. QS or SWS, AS or REMS, and IS times as percentages of TST	Behavioural Testing
(Fagioli & Salzarulo, 1982)	Sleep development	13	2 weeks to 11 months & 3 months	EEG, EOG, EMG	QS, Paradoxical sleep, Ambiguous sleep, Waking	
(Fagioli & Salzarulo, 1997)	Sleep development	12	9–47 weeks	Polygraphy	The range (difference between the value at the beginning of the QS episode and that at the trough), the trough latency (interval between QS onset and trough), and the rate of synchronisation (range/trough latency)	
(Fattinger et al., 2014)	Sleep development	11 (5:6)	2, 4, 6 & 9 months	Polysomnography	TST, % of NREM, % of REM, WASO, SE, SWS	
(Fifer et al., 2010)	Learning and memory	34	10-73 h of age	EEG	Eye movement response data	
(Figueiredo et al., 2016)	Sleep development	94 (54:40)	2 weeks, 3 & 6 months	The Infant Sleep Chronogram	Sleep hours, Awake hours, Awakenings, Latency to sleep, longest sleep period, 24 h period sleep	
(Franco et al., 2005)	Sleep deprivation	16 (10:6)	8 weeks old	Night-time polygraphy recording	NREM, REM, Wakefulness, Movement time	
(Franco et al., 2004)	Sleep deprivation	14 (4:10)	8 weeks old	EEG, EOG, ECG, EMG, Actigraph	NREM, REM, IS, Wakefulness, Movement time, SE	
(Fransson et al., 2009)	Sleep development	21 (9:12)	New-born	Functional MRI	Resting-state networks	
(Freudigman & Thoman, 1994)	Sleep development	31 (13:18)	2–5 days old	Motility Monitoring System	AS, QS, A-Q Transitional Sleep, S-W Transition, Wake, Out-of-the-crib	
(Freudigman & Thoman, 1998)	Sleep development	51 (23:28)	1–5 days old	Motility Monitoring System	AS, QS, A-Q Transitional Sleep, S-W Transition, Wake, Longest sleep period, Mean sleep period, Time in crib	
(Fukuda & Ishihara, 1997)	Sleep development	10 (7:3)	0–6 months	Sleep log	Sleep scored as 1 and wake scored as 0	
(Giganti et al., 2001)	Sleep development	12	2 weeks to 11 weeks & 3 months	Polygraph	QS, Paradoxical sleep, Ambiguous sleep, and Wakefulness	
(Goodlin-Jones et al., 2001)	Sleep development	80 (39:41)	3, 6, 9, & 12 months	Time-lapse video	Longest sleep period, % of time in AS, QS, wakefulness, & out of crib. No. and duration night-awakenings, Duration from vocalization to caregiver’s interaction. Time & duration of parent’s checking on sleeping infant, all parent interactions with wakeful infant during the night	
(Harper et al., 1981)	Sleep development	25 (16:9)	1 week, 1, 2, 3, 4, & 6 months	EEG, EMG, ECG	QS, AS, Waking and IS	
(Hayes et al., 2011)	Sleep development	120	6 weeks, 16 & 24 months	Day Diary, Sleep Habits Inventory	Diary: Sleeping, Awake & content, fussing, crying, feeding. Sleep Habits inventory: Bedtime, rise time, night waking, presence of sleep problems, ease of sleep onset, use of sleep aids	Infants/Toddler Symptom Checklist
(Henderson et al., 2020)	Sleep development	52	1, 3, 6, 9, & 12 months	Sleep diary, The composite Sleep Scale,	Sleep diary: frequency of night-waking, No. of S-W transitions over 24 h, Longest self-regulated sleep period duration, & TST/24 h. The Composite Sleep Scale: Avg. time of sleep onset or avg. bedtime, Total time slept at night, No. of waking/night, No. of nights waking/week, time awake/waking, Avg. weekly hours bedsharing (co-sleeping)	
(Hoppenbrouwers et al., 1988)	Sleep development	20 (11:9)	1 week, 1, 2, 3, 4, & 6 months	Polygraph	% TRT, % TST, No. of episodes during TRT, Mean & median duration of episode during TRT, Mean & median interval between onset of identical states, No. of epochs of sustained state, Mean duration of sustained states. Longest episode in AS, QS and Wake, AS-QS ratio. Sleep state transitions: Q–A, QS-Awake, Awake-QS, Awake-AS, AS-QS, AS- Awake	
(Horne et al., 2003)	Sleep development	2–3 months 21 (7:14) 5–6 months 16 (8:8)	2–3 & 5–6 months	Daytime polygraph	NREM, REM. Spindle frequency, duration, and density. %NREM with spindles, Arousal threshold	
(Horváth et al., 2018)	Learning and memory	45 (15:30)	86–122 days old	Polysomnography, Sleep and Naps Oxford Research Inventory	Night-time sleep, Daytime sleep, Awake time during night, Sleep time, % NREM2, % SWS, % REM, Sleep spindle density	Eye-tracking assessment
(Horváth et al., 2016)	Learning and memory	28 (14:16)	15–16 months	Sleep and Naps Oxford Research Inventory, Actigraph	Naptime, Wake time	Eye-tracking assessment, Oxford Communicative Development Inventory
(Horváth et al., 2015)	Learning and memory	38 (16:22)	16 months	Sleep and Naps Oxford Research Inventory,	Avg. sleep time, Avg. Nap time	Eye-tracking assessment, Oxford Communicative Development Inventory
(Hysing et al., 2014)	Sleep development	55,831 (28,507:27,324)	6, 18 months	Questionnaires	6 months: Sleep duration, Nocturnal awakenings, Easy to put to bed & falls asleep quickly, Co-sleeping after birth, Co-sleeping at 2 month, Co-sleeping at 4 months, Co-sleeping at 6 months 18 months: Sleep duration, Nocturnal awakenings	
(Hysing et al., 2016)	Sleep development	2,012 (1039:973)	2 years old	BISQ	TIB, Duration of wakefulness, Nocturnal settling time, Sleep duration, No. of nocturnal awakenings, is sleep an issue? Sleep onset latency, WASO	ASQ: Social-Emotional
(Iemura et al., 2016)	Sleep development	300	18 months	Japan Children's Study of Sleep Questionnaire	Wake time, Bedtime, No. of night-waking, Nap time, Daytime sleep, TST, SE, TIB, Total nap time	Neurobehavioral observation, MCHAT, KINDER Infant Development Scales
(Jacklin et al., 1980)	Sleep development	161 6 months = 17, 9 months = 19, 12 months = 16, 18 months = 73, 26 months = 36	6, 9, 12, 18, 26 months old	Sleep diary	Longest period of sleep, longest period of wakefulness, Total sleep, No. of sleep-wakefulness transitions	
(Jenni et al., 2004)	Sleep development	11(5:6)	2 weeks, 2, 4, 6, & 9 months old	All-night home polysomnography	TRT, Total sleep of first 418.7 min of TRT, Duration and % SE, %WASO, Duration and % of QS/NREM, AS/REM, % IS, Movement time, Duration of sleep cycles, No. of cycles per subject	
(Kärki et al., 2020)	Sleep development	72 (34:38)	8 months old	Overnight home polysomnography, Questionnaire	Questionnaire: regularity of bedtime routine, typical time of falling asleep, No. & duration of daytime naps. PSG: %REM, %N1, %N2, %N3, %NREM, Wakefulness, TST, Sum of awaking & arousal index, %SE, WASO, Sleep onset latency, REM onset latency	
(Karki et al., 2019)	Sleep development	85 (43:42)	1 month old	Overnight home polysomnography	%REM, %NREM, Wakefulness, TST, Sum of awaking and arousal index, %SE, WASO, No. of times awake	
(Kocevska et al., 2018)	Sleep patterns	6,808	2 years old	Questionnaires	Bedtime, Wake time, Amount of daytime sleep, Total sleep duration	
(Korotchikova et al., 2016)	Sleep development	80 (42:38)	Within 36 hs after birth	Continuous Video-EEG	AS, QS, IS, Wakefulness, Sleep–wake cycle	
(Korte et al., 2004)	Sleep development	57 (32:25)	Birth—6 days old	Actigraph	Daytime sleep, night-time sleep, 24 h sleep	
(Ktonas et al., 1995)	Sleep development	28 2–6 weeks = 10, 7–14 weeks = 10, 16–48 weeks = 8	2–6, 7–14, 16–48 weeks	Whole night polysomnography	Longest phase of QS, Mean, SD, and range of the longest period of QS	
(Lampl & Johnson, 2011)	Sleep and growth	23 (9:14)	4–17 months old	Sleep records	No. of sleep bouts, No. of hours per bout, Total sleep hours	
(Liefting et al., 1994)	Sleep development	23	4–47 weeks old	Polygraph	Waking, Paradoxical sleep, QS, SWS, Tonic EMG, EMG instability	
(Louis et al., 1997)	Sleep development	15 (7:8)	3, 6, 9, 12, 18, & 24 months	24 h EEG	Duration and % of Wakefulness, REM, NREM stages 1, 2, 3, IS & SWS. TRT, SEI	
(Louis et al., 1992)	Sleep development	12 (6:6)	1.5–3 months, 4.5-6 months	Overnight polygraphy records	IS, QS/NREM, AS/REM, Sleep spindles: Location, Density, Frequency, Amplitude, Asymmetry, Asynchrony	
(Lukowski & Milojevich, 2013)	Sleep development	25	10 months	BISQ	Night-time sleep duration, Frequency of night-waking, Daytime sleep duration, % of sleep obtained at night	Elicited Imitation
(Matsuoka et al., 1991)	Sleep development	33 (12:21)	8 days—4 months	Sleep records	Sleep, Wakefulness	
(Miano et al., 2011)	Sleep development	11 (7:4)	5–16 months	Daytime polygraph	Sleep period time, TST, Sleep latency, REM latency, SE, WASO (%), N1 (%), N2 (%), N3 (%), REM (%), CAP rate (%), CAP rate S1, S2, S3, CAP time, A1 (%), A2 (%), A3 (%), A1 index, A2 index, A3 index, A mean duration, B mean duration	
(Mindell et al., 2012)	Sleep development	92 (36:56)	3–18 months	BISQ	No. of night-waking, longest continuous sleep period, Total night-time sleep, Sleep onset latency, Number of daytime naps, how often wakes in own bed, consider sleep a problem	
(Mindell et al., 2017)	Sleep and socio-emotional development	117	6, 12, & 18 months	BISQ	Bedtime, Sleep onset latency, No. of night-waking, longest continuous sleep period, TST in a 24 h period	ITSEA
(Nakagawa et al., 2016)	Sleep patterns	50	1.5 years	Actigraphy	Naps during week, Nap duration, Bedtime, Wake Time, Sleep duration	
(Navelet et al., 1982)	Sleep development	42 (18:24)	1–7 months	Polygraph	Total sleep recording, Duration and % of AS, QS, Transitional Sleep, Wakefulness, TST	
(Parmelee et al., 1961)	Sleep development	75 (34:41)	1–3 days	Day diary	Avg. TST, Avg. longest sleeping period	
(Peirano et al., 1993)	Sleep development	48	1–54 weeks	Polygraph	Qs, Paradoxical sleep, Ambiguous sleep, Waking	
(Pennestri et al., 2020)	Sleep development	44 (22:22)	6 months	Sleep diary	Longest sleep duration, No. of nocturnal awakenings, % of 6 h. % of 8 h	
(Pennestri et al., 2018)	Sleep development	6 months = 388 (206:182) 12 months = 369 (193:176)	6, 12 months	Questionnaires	Slept through the night- 6 h, Slept through the night-8 h	Bayley Scales of Infant Development-II
(Pisch et al., 2019)	Learning and memory	40 (19:21)	4, 6, 8, & 10 months	BISQ, Actigraph	Night sleep time, WASO, Avg. night waking frequency, Avg. daytime sleep time	Eye-tracking tasks assessing memory
(Ramamurthy et al., 2012)	Sleep development	4602	Birth-12 months	BISQ	Infants’ bedtime, No. of night-waking, Duration of night-waking, No. of naps, Duration of daytime sleep, TST, Sleep latency over 30 min	
(Ribner et al., 2019)	Screen exposure and sleep	419 (218:211)	4 months	BISQ	Night-time sleep, Daytime sleep, Total sleep, No. of wakes per night	IBQ very short form questionnaire
(Sankupellay et al., 2011)	Sleep development	34 (16:18)	2 weeks, 3, 6, 12, & 24 months	Full night polysomnography	Duration of TRT, TST, Awake time, QS/NREM, NREM-N1, NREM-N2, NREM-N3, NREM-N, Movement time. %TST: SE, QS/NREM, NREM-N1, NREM-N2, NREM-N3, NREM-N, AS/REM	
(Satomaa et al., 2020)	Sleep development	56 (24:32)	8 months	Overnight polysomnography	TIB, TST, NREM, %N1, %N2, %N3, Artifact free NREM time (FFT), %REM, Awakening Index, Arousal Index	Bayley Scales of Infant Development-III
(Scher, 1991)	Sleep development	118	3, 6, 9, & 12 months	Sleep Questionnaire	Night waking, No. of interrupted nights/week, Mins to settle to sleep at bedtime, Mins to settle to sleep after awakening, No. of hours of sleep at night, No. of hours of sleep during the day	
(Scher, 2005b)	Sleep development	50 (26:24)	10 months	Sleep Questionnaire	No. of interrupted nights, No. of awakenings/night, Avg. time spent awake, Sleep onset time, Latency to fall asleep, Total sleep duration	
(Scher & Cohen, 2005)	Sleep and gross motor development	107 (57:50)	5–8 months	Sleep Habit Questionnaire, Infant Sleep questionnaire	Sleep Habit Questionnaire: No. of interrupted nights, No. of awakenings/night, Avg. time spent awake. Infant sleep questionnaire: Sleep problem score	Gross Motor Checklist
(Scher, 2005a)	Sleep and gross motor development	59	8 months	Sleep Questionnaire, Actigraphy	Sleep Questionnaire: Night-waking Index, Sleep Schedule. Actigraphy: Sleep onset time, Duration of sleep, % of activity/minute of sleep, No. of transitions from sleep–wake, longest continuous sleep period without identified wake, SE, Wake	Gross Motor Checklist
(Scher & Cohen, 2015a)	Sleep and gross motor development	28 (12:16)	4—11 months, visits every 2–3 weeks	Actigraphy, Sleep diary	Actigraphy: No of long wake episodes, Hour of sleep onset, Mins of entire sleep period, SE. Sleep diary: Bedtime, Night waking episodes (No. and duration)	Motor milestone diary, Motor observations, The Infant Characteristic Questionnaire
(Scher et al., 1995)	Sleep development	33	1–3 days old	Sleep EEG	AS, QS, IS, REMS/minute, Cycle length, EEG correlation, Arousal, Movements, % low voltage irregular active sleep segment, % Trace alternant, Delta, Theta, Alpha, Beta	At 2 years: Bayley Motor and Mental Performance tests, Vineland Social Maturity Scales, Carey Temperament Questionnaire, Parental Report
(Seehagen et al., 2015)	Learning and memory	Exp1:120 (60:60) EXP 2:96	6 & 12 months	Actigraphy, Sleep log	Amount of time asleep during nap	Memory task
(Simon et al., 2016)	Sleep and language	37	6.5 months	Nap Polysomnography, Sleep Questionnaire	TST, WASO, Sleep latency, SE, NREM Stages 1, 2, and 3, REM, SWS, Theta, Alpha, Sigma, Beta	Artificial Speech stream
(Spruyt et al., 2008a)	Sleep development	20 (13:7)	Monthly from 1–12 months	Sleep diaries, Actigraphy	% 24 h sleep duration, % Nocturnal sleep, % Diurnal sleep	Early Infant Temperament Questionnaire (ITQ) at 3 months, Revised ITQ at 6 & 12 months, Bayley Scales of Infant Development-II at 12 months
(Sterman et al., 1977)	Sleep development	10 (4:6)	4–7 days, 1, 2, 3, 4, & 6 months	Polygraph	AS, QS, Awake, Transitions	
(Sun et al., , 2018b)	Sleep and face processing	52	12 months	Actigraphy	TST, Daytime sleep, Night-time sleep, Sleep onset latency, WASO, Night-time SE, Circadian Rhythm activity	Eye-tracking face processing task
(Sun, et al., 2018a)	Sleep and development	590 (321:269)	2–30 months	Chinese version of BISQ	Night sleep duration, Daytime sleep duration, Total sleep duration, Duration of night-time awakenings	Chinese version of Bayley Scales of Infant Development—I, The Psychomotor Developmental Index
(Sun et al., 2016)	Sleep and face processing	49 (25:24)	6 months	BISQ	Night sleep duration, Daytime sleep duration, Total sleep duration, Mean night waking duration, Night wake frequency, % Night sleep	Eye-tracking preferential looking behaviour task
(Thoman & McDowell, 1989)	Sleep development	20	2, 3, 4, & 5 weeks old	Sensor mattress, Sleep form	Cyclicity Analysis	
(Tikotzky et al., 2010b)	Sleep and growth	96 (62:34)	6 months	BISQ, Actigraph	BISQ: Sleep onset time, Nocturnal sleep duration, Daytime sleep duration, No. of night-waking, Sleep position. Actigraph: Total sleep period, True sleep time, Sleep %, No. of night-waking	Developmental Questionnaires, Growth Measures
(Tikotzky et al., 2015)	Sleep development	56 3 months, 54 6 months	3 & 6 months	BISQ, Actigraph, Sleep diary	BISQ: Night-time involvement, Actigraph: Sleep minutes, SE, No. of long wake episodes. Sleep Diary: Infant daytime sleep duration, Infant no. of night-waking,	
(Wielek et al., 2019)	Sleep development	42 (27:15)	2 & 5 weeks old	Polysomnography	QS/NREM, AS/REM, Wake, Movement time, Transitional sleep, Power spectral density, Entropy measure	
(Wooding et al., 1990)	Sleep patterns	874 (432:428)	1–12 months	Sleep diary and Questionnaires	TST over 24 h, Night-time sleeping, Daytime sleeping, Uninterrupted night sleeping, Sleeping, Waking & Settling times, Bedtime routines	
(Yoshida et al., 2015)	Sleep development	34 (17:17)	3 & 4 months	Actigraph, EEG, Sleep log	Actigraph: Wakefulness, Light sleep, Deep sleep, EEG: Total sleep, Wake, REM, NREM stages 1–2, SWS, No. of sleep cycles	
(Zhou et al., 2015)	Sleep development	899 (475:424)	3, 6, 9, 12, 18, & 24	BISQ	Avg. night sleep, Avg. Day sleep, Total daily sleep duration,	Growth measures

*AS* Active Sleep, *QS* Active Sleep, *SE* Sleep Efficiency, *BISQ* Brief Infant Sleep Questionnaire, *EEG* Electroencephalogram, *SWS* Slow wave sleep, *TIB* Time in Bed, *TST* Total Sleep Time, *WASO* Wake after sleep onset, *%* percentage, *No.* number, *CAP* Cyclic Alternating Pattern, *PSG* Polysomnography, *Q–A* Quiet to Active, *A-Q* Active to Quiet

**Table 3:** Weighted means of daytime sleep, night-time sleep, and total sleep time over a 24 h period

Age (Months)	1	2	3	4	5	6	7	8	9	10	11	12	18	24
Daytime Sleep (h)	6.4	2.9	4.9	3.5	–	2.5	–	2.9	3.2	2.8	–	2.7	1.5	–
Night-time Sleep (h)	6.7	8.2	8.8	9.5	9.3	10.7	11.7	9.5	10	10	9.9	9.5	11.3	7.4
TST/24 h period (h)	13.3	15.7	12.2	13.6	–	13.1	–	–	11.7	–	–	12.2	12.7	13

*TST* Total Sleep Time, *h* hour. Daytime weighted means calculated from (Adams et al., 2019; Blair et al., 2012; Figueiredo et al., 2016; Pisch et al., 2019; Ribner et al., 2019; Scher, 1991; Sun et al., 2018a, 2018b; Tikotzky et al., 2010b). Night-time weighted means calculated from (Adams et al., 2019; Blair et al., 2012; Fattinger et al., 2014; Figueiredo et al., 2016; Nakagawa et al., 2016; Navelet et al., 1982; Pisch et al., 2019; Ribner et al., 2019; Sankupellay et al., 2011; Scher, 1991, 2005a, 2005b; Scher & Cohen, 2015a; Sun et al., 2018a, 2018b; Sun et al., 2016; Tikotzky et al., 2010b; Tikotzky et al., 2015) TST/24 h period weighted means calculated from (Bernier et al., 2010; Blair et al., 2012; Figueiredo et al., 2016; Hysing et al., 2016; Kocevska et al., 2018; Mindell et al., 2017; Nakagawa et al., 2016; Ribner et al., 2019; Sun et al., 2018a, 2018b; Sun et al., 2016)

### Daytime Sleep

Eight studies provided daytime sleep data (see Table 3). Daytime sleep decreased from 6.5 h at 1 month to 1.5 h at 18 months (Acebo et al., 2005; Adams et al., 2019). Daytime sleep is essential for learning and memory consolidation (Cheour et al., 2002; Fifer et al., 2010; Horváth et al., 2015, 2016, 2018; Lukowski & Milojevich, 2013; Seehagen et al., 2015; Simon et al., 2016). One study suggested controlling the timing and duration of afternoon naps in 18-month-old children to promote earlier sleep onset and longer night-time sleep (Nakagawa et al., 2016). No data were available for 13–17 and 19 – 24 months.

### Night-time Sleep

Data for night-time sleep were collected from 17 studies (See Table 3). Night-time sleep increased between 1–7 months (6.7 h to 11.7 h) and decreased at eight months (9.5 h). Sleep increased again at nine months (10 h) and decreased at 24 months (7.4 h). High amounts of night-awakenings were observed between 17–20 weeks (Giganti et al., 2001). Night-awakening decreased between 6–18 months (Blair et al., 2012). Infants who were crawling between 5–8 months of age had more disrupted sleep compared to their pre-crawling peers (Scher & Cohen, 2005; Scher, 2005a). At six months (n = 388) of age, 37.6% of infants slept for less than six consecutive hours at night, while 43% of infants slept for eight consecutive hours. At 12 months (n = 369), 72.4% of infants slept six consecutive hours at night and 56.6% slept eight consecutive hours (Pennestri et al., 2018). Occasional sleeping throughout the night did not prevent future night-awakenings (Pennestri et al., 2020). Swaddling resulted in decreased night-awakenings (Franco et al., 2005).

Breastfed infants had significantly more night-awakenings compared to formula-fed infants (mean (SD) 1.63 (1.24) vs 0.94 (0.87), *p* = 0.003 (Mindell et al., 2012)) but this resolved within the first year (Eaton-Evans & Dugdale, 1988; Mindell et al., 2012; Tikotzky et al., 2010b), and infants who were nursed back to sleep in the first year also had more night-awakenings (Ramamurthy et al., 2012). Co-sleeping was reported as a risk factor for shorter sleep duration and more frequent night-awakenings from 6–18 months (Hysing et al., 2014). One hour of exposure to electronic screen-based media at four months resulted in 13 min less nocturnal sleep (Ribner et al., 2019).

### Total Sleep Time

Total sleep time over a 24 h period was available from 10 studies. The average TST was 13.13 h at one month, 15.7 h at two months, 12.2 h at one year, and 13 h at 24 months (Bamford et al., 1990; Figueiredo et al., 2016; Jacklin et al., 1980; Wooding et al., 1990). The number of sleep episodes decreased from 6.1 at two weeks to 5.2 at six months (Figueiredo et al., 2016). The duration of time awake at two weeks increased from 8.7 h to 10 h at six months, while the longest period of sleep increased from 3.2 h at two weeks to 5.6 h at six months (Figueiredo et al., 2016). Sleep efficiency (ratio of TST to time spent in bed) and periodicity increased by 12 months, with a shift towards night sleep, over daytime sleep (Louis et al., 1997; Wooding et al., 1990).

Summer born infants were reported to have shorter TST (Karki et al., 2019; Kärki et al., 2020). Parental presence at bedtime, frequency of night-awakenings, and less TST at one month of age predicted poor sleep at six and twelve months (Henderson et al., 2020). Greater parental involvement in both daytime and night-time care at three months predicted more consolidated maternal and infant sleep at six months (Tikotzky et al., 2015).

### Sleep States

The duration of QS was higher on the day of birth compared to one day later in 19 infants born vaginally and 17 born by emergency Caesarean-section (CS) after a period of labour. This may be a temporary response to the stress of labour (Carroll et al., 1999). From birth, AS occupies a greater percentage of TST (Ellingson & Peters, 1980; Hoppenbrouwers et al., 1988; Korotchikova et al., 2016), with QS occupying less than half and IS occupying 5–13% of TST (Ellingson & Peters, 1980). Sleep in neonates begins in AS rather than QS/NREM sleep (Ashton, 1971; Ellingson & Peters, 1980; Hoppenbrouwers et al., 1988). As sleep matures over the first year, AS-onset gives way to QS-onset, and the percentage of TST spent in AS (between 50–80% to less than 50%) and IS decreases. The percentage of TST spent in QS increases to approximately 35–50% in the first year (Anders, 1978; Dittrichová et al., 1972; Ellingson & Peters, 1980; Fagioli & Salzarulo, 1997; Fattinger et al., 2014; Hoppenbrouwers et al., 1988; Jenni et al., 2004). After two months, AS and QS become REM sleep and NREM sleep, and the stages of NREM sleep begin to appear (Sankupellay et al., 2011).

By six months, all sleep should begin with NREM onset (Ellingson & Peters, 1980). The increase in NREM sleep duration is associated with the appearance of slow-wave sleep (SWS), or NREM stage 3, showing the maturational restructuring of sleep (Ktonas et al., 1995; Peirano et al., 1993). A potential increase in the proportion of SWS was seen in exclusively breast-fed 3–4 month old infants (Yoshida et al., 2015).

### Electroencephalogram Characteristics of Sleep

Within 6–12 h of birth, the EEG of healthy term neonates shows continuous, symmetrical, and synchronous activity at amplitudes of approximately 15–150 µV across all sleep states, with well-developed sleep–wake cycles (SWC) (Korotchikova et al., 2016). Markers of sleep homeostasis (low-frequency delta activity and declining theta activity throughout the night) are present in the first months (Jenni et al., 2004). REM or AS in healthy term neonates is seen on the EEG as continuous irregular, low-voltage activity with amplitudes of 15–35 µV at frequencies between 5–8 Hz. NREM/QS is continuous and synchronous with higher amplitudes of 50–150 µV.

*Tracé alternant* pattern is the most frequent EEG pattern detected during QS in healthy term neonates and is present from birth. *Tracé alternant* is characterized by bursts of high amplitude slow-wave activity (SWA) with intermixed faster frequencies, separated by lower amplitude mixed frequency activity (Eiselt et al., 2001; Ellingson & Peters, 1980) and begins to disappear rapidly from the EEG from 2 weeks of age and is not seen after six weeks (Bes et al., 1988; Ellingson & Peters, 1980).

Sleep spindles are a hallmark of stage 2 NREM sleep. Sleep spindles should be present in NREM stage 2 on EEG recording by three months (Corsi-Cabrera et al., 2020; Ellingson & Peters, 1980; Horne et al., 2003; Jenni et al., 2004; Louis et al., 1992; Navelet et al., 1982; Sankupellay et al., 2011; Sterman et al., 1977). Spindles and SWS are believed to stimulate the development of thalamocortical networks by supplying endogenous neural signals with repetitive and co-ordinated activity (Jenni et al., 2004). The density of sleep spindles, and NREM proportions with sleep spindles was more frequent when the infant was placed supine rather than prone (Horne et al., 2003). In stage 2 NREM sleep, another characteristic waveform called the K complex can appear by as early as 5 months and although its exact function during sleep is not precisely known it is believed to play a role in sleep promotion and arousal. Vertex sharp waves usually appear in the EEG at the age of 5–6 months. Between 5–16 months, Cyclic Alternating Pattern (CAP) is present during NREM sleep as a physiologic oscillating pattern. CAP is important for the build-up and maintenance of sleep. It has 2 phases, A and B, with A having three subtypes based on EEG patterns. Subtype A1 is based on the prevalance of EEG synchrony, subtype A3 is based on the prevalence of EEG desynchronization, and subtype A2 is a combination of both. Miano et al. showed that a decreased frequency of the CAP A1 subtype may indicate maturation of the arousal system (Miano et al., 2011).

### Sleep and Neurodevelopment

Increasing age leads to increased ability to self-soothe after night-time awakening (Goodlin-Jones et al., 2001). At 12 months, decreased TST during the day correlated with better emotional regulation, as measured using the Behaviour Rating Scale subtest of the Bayley Scales of Infant Development II (Spruyt et al., 2008a). Infants and young children with a higher percentage of night-time sleep had more advanced executive function (Bernier et al., 2010). Fewer night-awakenings after sleep onset were identified as markers for better performance on a working memory task (Pisch et al., 2019). Sleep onset after 22:00 h and longer daytime nap durations were associated with poor neurodevelopmental outcome (Iemura et al., 2016). REM sleep storms (intense REM bursts with increased eye and facial movements) are considered normal in the first five weeks, but if present at six months, were associated with significantly lower scores on the Bayley Scale of Infant Development at one year (Becker & Thoman, 1981). Frequent night-awakening was associated with poor cognitive function in young children aged 12–30 months (Sun et al., 2018a, 2018b). At eight months, better performance on the Bayley-III psychomotor development evaluation was associated with slow-wave activity total power on EEG (Satomaa et al., 2020).

Hayes et al. reported that sleep–wake patterns and temperament were stable over the first 24 months (Hayes et al., 2011).However, other studies reported short sleep duration, night-awakenings, sleep onset problems, and later bedtime were associated with social and emotional problems (Hysing et al., 2016; Mindell et al., 2017). Longer TST and better sleep quality were associated with social learning at six and twelve months (Sun et al., 2016, 2018a). Greater motor activity during sleep and fragmented night sleep was associated with lower mental developmental index scores on the Bayley Scales (Scher, 2005b). The emergence of motor skills was associated with sleep disruption (Atun-Einy & Scher, 2016; Scher & Cohen, 2015b).

### Sleep and Growth

Between 4–17 weeks, sleep was temporally related to growth (Lampl & Johnson, 2011). Short sleep duration was linked to weight gain and obesity in young children (Tikotzky et al., 2010b). Infants who slept less than 12 h per day at three months had higher body mass index (BMI) and shorter body length (Zhou et al., 2015). The search returned only these studies investigating the relationship between sleep and growth hormone secretion suggesting there is a gap in the literature.

### Sleep and Mode of Delivery

Two studies reported the influence of mode of delivery on sleep. Neonates delivered vaginally and by emergency CS (following a period of labour) showed more sleep periods in the daytime compared to neonates born by elective CS (Korte et al., 2004). A significant decrease in AS and increase of QS in the EEG recordings of neonates delivered by elective CS was observed compared to neonates born vaginally or by emergency CS (Korotchikova et al., 2016). One study reported that mode of delivery did not affect sleep (Scher et al., 1995).

### Sleep Deprivation

Neonates spent more time asleep after a period of sleep deprivation (Anders & Roffwarg, 1973). Short-term sleep deprivation in 6–18 week old infants was associated with development of obstructive sleep apnoea and a significant increase in the threshold for auditory arousal in sleep immediately after sleep deprivation (Franco et al., 2004).

## Discussion

Rapid changes occur in sleep architecture over the first year and this review highlights the importance of sleep for neurodevelopment. Over the first two years, AS decreases while QS increases. The number of sleep episodes, TST, and night-awakenings all decrease with age, while the longest periods of sleep and wakefulness increase. This decrease in TST is not linear, as shown in Fig. 2. This may be due to the attainment of different developmental milestones, such as crawling and pull-to-stand as mentioned in this review (Atun-Einy & Scher, 2016; Scher, 2005a). Parents often report a period of unsettled or disrupted sleep around specific developmental milestones and some experts refer to this as sleep regression (Foley, 2020).

There are inconsistencies in how sleep variables are reported in the literature. This makes comparisons between studies complex in many cases. Serious ethical concerns about studies involving sleep deprivation in infants also exist. Ander and Roffwarg used foundling infants (infants left by their parents at hospitals/churches etc. (The Foundling Museum, 2020)) in their study (Anders & Roffwarg, 1973), which raises ethical concerns. Sleep depriving 6–18 week old typically developing infants (Franco et al., 2004) also raises ethical concerns.

Some sleep studies were conducted during daytime naps, and others during nocturnal sleep. This can make it challenging to compare sleep variables between studies as daytime records are often shorter and daytime naps have disproportionately less REM as development progresses (Coons & Guilleminault, 1984). This can lead to differences in reference values (Coons & Guilleminault, 1984; Ellingson & Peters, 1980; Figueiredo et al., 2016; Louis et al., 1992).

A common limitation throughout the studies was sample size. Studies acknowledged that a small sample size might have resulted in large effect sizes being detected with a possibility of type II error (Atun-Einy & Scher, 2016; Eiselt et al., 2001; Fattinger et al., 2014; Franco et al., 2004; Jenni et al., 2004; Korotchikova et al., 2009; Louis et al., 1992; Scher & Cohen, 2015a; Spruyt et al., 2008b; Tikotzky et al., 2015). The specific population studied may also be a limitation (Franco et al., 2004; Ramamurthy et al., 2012; Tikotzky et al., 2010a, 2015), with an unevenly distributed age (Franco et al., 2004), parents from medical professions (Franco et al., 2004), middle-to-high-income families in Israel (Tikotzky et al., 2015) or highly educated parents and firstborn infants (Tikotzky et al., 2010a). Ramamurthy et al*.* completed a study which was solely conducted online and was therefore only available to those with internet access (Ramamurthy et al., 2012).

A limitation observed in EEG studies was that some sleep recordings did not capture a full SWC, influencing the interpretation and analysis of the different sleep stages (Dittrichova, 1966; Korotchikova et al., 2016). Korotchikova et al*.* observed that a complete SWC was not captured for over 50% of their neonatal cohort, despite having an hour of EEG recording for each neonate. Based on their data, the authors recommended sleep EEG recordings of 150 min to ensure capture of a complete SWC (Korotchikova et al., 2016). The number of channels used for the EEG recording and the frequency of EEG recordings was a noted limitation in some studies (Sankupellay et al., 2011; Yoshida et al., 2015). Future studies should comprehensively characterise sleep EEG biomarkers over the first two years of life and correlate them with neurodevelopmental outcomes (Ventura et al., 2022).

One study using actigraphy raised concerns about reliability (Scher & Cohen, 2015a) in infant and young children studies, with another study arguing that actigraphy should be accompanied by a sleep diary (Bernier et al., 2010). Scher et al. highlighted they captured three nights of actigraphy instead of the recommended five nights (Scher & Cohen, 2015a).

The search terms used in this paper may have limited the scope of this review and missed some elements of sleep development, for example K-Complexes and vertex waves. In a review on sleep neurophysiology and maturation in infants and young children, Dan & Boyd discussed the development of K complexes and vertex sharp waves during sleep (Dan & Boyd, 2006). K-Complexes are usually present between 5–6 months of age and are well established by 18 months (Grigg-Damberger et al., 2007; Metcalf et al., 1971; Verma & Baisakhiya, 2021). However, the search strategy used in this review did not capture any studies studying these features. This should be taken into consideration when reading this review.

A strength of this narrative review was that the search strategy was developed using the PRISMA guidelines, resulting in the search being completed in a systematic way with predefined inclusion and exclusion criteria. The narrative review allowed us to look at all methods used for assessing sleep and allowed for a wider scope for inclusion of literature in this review, as systematic reviews are often restrictive with their research question.

## Conclusion

Despite a considerable volume of research on sleep in the first year, research on the impact of sleep on neurodevelopment is lacking. Infants and young children spend most of the time asleep, with QS increasing and AS decreasing during maturation (Figueiredo et al., 2016). Studies highlighted the importance of sleep for learning and memory. While it is difficult to investigate the impact of sleep disruption and sleep deprivation on neurodevelopment, infants admitted for long periods in the NICU may shed some light on the effect of sleep disruption in early life on long-term outcome (Levy et al., 2017; van den Hoogen et al., 2017). However, this cohort may have sleep disruption and poor neurodevelopmental outcomes due to underlying pathology. An example of a population which may be more suited to longitudinal study of sleep disruption and neurodevelopmental outcome are infants and young children with moderate-to-severe eczema. These young children are typically developing but have disrupted sleep due to itch (Camfferman et al., 2010).

A lack of literature documenting sleep in healthy 7–24-month-old children was found during this review. As a result, gaps in knowledge about the maturation of sleep in the first two years of age exist. Longitudinal studies require a significant commitment, and monthly appointments over a 2–3 year period may be inconvenient for participating parents. To fill in these knowledge gaps, future studies should consider assessing sleep in the second year of life, as this continues to be an important period of plasticity and development (Cusick & Georgieff, 2017).

Uniformity in scoring sleep stages is recommended, as well as a consensus on whether night-time or daytime sleep studies are best. Alternatively, it may be necessary to compare daytime sleep studies with other daytime studies and similar for nocturnal studies to ensure consistent and reliable reference values. Given the importance of sleep for neurodevelopment, future longitudinal studies may need to focus more closely on specific time points in the first two years and use EEG to identify normal sleep neuro-biomarkers.

## Data Availability

Not Applicable.
